# Evidence toward the potential absence of relationship between temporal and spatial heartbeats perception

**DOI:** 10.1038/s41598-021-90334-z

**Published:** 2021-05-24

**Authors:** Betka Sophie, Łukowska Marta, Silva Marta, King Joshua, Garfinkel Sarah, Critchley Hugo

**Affiliations:** 1 Clinical Imaging Science Centre, Brighton and Sussex Medical School, Sussex, Brighton, BN1 9RY UK; 2 Laboratory of Cognitive Neuroscience, Brain Mind Institute and Center for Neuroprosthetics, Faculty of Life Sciences, Swiss Federal Institute of Technology, (EPFL), 1202 Geneva, Switzerland; 3 Department of Clinical Neuroscience, Faculty of Medicine, University of Geneva, 1211 Geneva, Switzerland; 4 Consciousness Lab, Institute of Psychology, Jagiellonian University, Kraków, Poland; 5 Sackler Centre for Consciousness Science, University of Sussex, Sussex, BN1 9QJ UK

**Keywords:** Consciousness, Perception, Emotion, Insula, Autonomic nervous system

## Abstract

Many interoceptive tasks (i.e. measuring the sensitivity to bodily signals) are based upon heartbeats perception. However, the temporal perception of heartbeats—*when* heartbeats are felt—varies among individuals. Moreover, the spatial perception of heartbeats—*where on the body* heartbeats are felt—has not been characterized in relation to temporal. This study used a multi-interval heartbeat discrimination task in which participants judged the timing of their own heartbeats in relation to external tones. The perception of heartbeats in both time and spatial domains, and relationship between these domains was investigated. Heartbeat perception occurred on average ~ 250 ms after the ECG R-wave, most frequently sampled from the left part of the chest. Participants’ confidence in discriminating the timing of heartbeats from external tones was maximal at 0 ms (tone played at R-wave). Higher confidence was related to reduced dispersion of sampling locations, but Bayesian statistics indicated the absence of relationship between temporal and spatial heartbeats perception. Finally, the spatial precision of heartbeat perception was related to state-anxiety scores, yet largely independent of cardiovascular parameters. This investigation of heartbeat perception provides fresh insights concerning interoceptive signals that contribute to emotion, cognition and behaviour.

## Introduction

Interoception is the sense of the internal state of the body, and includes the perception of bodily signals coming from the viscera or glands^1, 2^. Interoception conveys and represents essential physiological information concerning health (including internal sensations of somatic pain and bodily temperature), and gives rise to motivational feelings including experience of thirst and hunger. Neurally, afferent signals travel via vagus nerve and spinal pathways (notably the Lamina I spinothalamocortical tract) to brainstem and thalamus, and are then relayed onto cortex, particularly the posterior and mid insula^3^. A further projection into anterior insula is proposed to give rise to an integrated representation of these bodily signals that are accessible to conscious appraisal and serves as a dynamic substrate for subjective feelings^4^.

Interoceptive signals, for the most part, inform automatic unconscious homeostatic reflexes^5^. People vary in their capacity for conscious access to interoceptive sensations, and these individual differences are considered relevant to the experience of emotions and vulnerability to pathological psychological and somatic symptoms^6^. Consequently, research has focused on the measurement of individual differences in interoception, using behavioral tasks through which interoceptive accuracy can be quantified from performance^7^. Most widely, such tasks target the perception of cardiac motion (heartbeats) at rest: Heartbeats are clear and discrete events, which are easily measurable^8^ and relevant—the changing strength and timing of heartbeats are signatures of changing physiological arousal that accompany emotions, exercise, injury, or illness. Hence, tests of heartbeat perception dominate such tasks, aiming to provide a baseline metric of interoceptive sensitivity. There has been less interest in detailing the physiological origins of the perception of heartbeats, which accompanies the ejection of blood from the heart into the aorta at ventricular systole, and includes physical changes within the vessels, chest and body (including the somatosensory, quasi-interoceptive, percussion of heart motion against of the inner chest wall). Some cardiac interoception tasks rely on the participant discriminating the timing of their own heartbeats relative to a phasic external stimulus, such as an auditory tone or flashing light. This approach raises possible confounds since different people might perceive heartbeats through different sensory channels, and hence vary in when and where they perceive their own heartbeats relative to other people^9–13^. One channel of interoceptive cardiac information comes from the phasic firing of specialized arterial (aortic and carotid) baroreceptors, as blood ejected from the heart stretches the vessel walls^14^. Some earlier researchers estimated, across individuals, the average delay between the ventricular contraction, peak baroreceptors activation and the heartbeat sensation to be approximately 150ms^15^. More systematic investigations of the temporal perception of heartbeat sensations indicate that people judge external auditory tones to be most simultaneous with heartbeat sensations if presented between 100 and 300 ms after the electrocardiogram (ECG) R-wave, the signature of myocardial electrical depolarization triggering ventricular contraction. Typically, the mean or the median of chosen temporal intervals is qualified as the temporal position of heartbeat sensation which lies between 228 and 288 ms after the R-wave^9, 12, 13, 16, 17^.

However, such results do not only represent ‘pure’ interoceptive information conveyed to the brain via the vagal and spinal afferent pathways. Indeed, despite full denervation of the heart and aortic arch, some heart transplant recipients may accurately feel their heartbeats^18^. Similarly, on a heartbeat detection task, a patient with both an extracorporeal left ventricular assist device and an endogenous heart was following artificial pump-beats (via abdomen somatosensory feedback) rather than his actual endogenous heartbeats^19^. Finally, a patient with extensive bilateral damage to the insula and anterior cingulate cortex—structures underlying interoceptive processes^20^—showed preserved interoceptive accuracy after the bolus administration of isoproterenol^21^. Only after anaesthetising the patient’s chest in the region of maximal heartbeat sensation, interoceptive awareness was impaired^21^. Together, these findings suggest that the somatosensory pathway also contributes to heartbeat sensations.

The spatial location of heartbeat sensations has not been studied as extensively as the temporal aspect. Khalsa and colleagues asked participants to trace on a manikin template (representing their own body) the location of their heartbeat sensations. During low arousal states, participants mostly felt their heartbeats in the lower left chest. Some participants also reported heartbeat sensations in the head, neck, belly and arms^21–24^. Good heartbeat perceivers, based on how accurately they performed the heartbeat detection ‘counting’ task (in which the reported number of ‘felt’ heartbeats, counted over different time periods are compared to veridical heartbeats, measured using ECG) report more spontaneous sensations (SPS; e.g. tickling, tingling or even warming sensations) in the hands than poor heartbeat perceivers^25^. The time interval from the ECG R wave to the finger pulse (pulse transit time) is typically estimated to ~ 250ms^26^. However, microneurography reveals pulse-triggered firing of mechanoreceptors in the fingers to occur as early as 200 ms after ECG R-wave^27^. Nevertheless, the relationship between temporal and spatial locations of heartbeat sensations has not yet been explored.

Interestingly, interoceptive abilities are likely impaired across different psychological conditions associated with affective symptoms. Indeed, alexithymia—classically defined as the difficulties to identify and describe one’s feelings—has been proposed to be caused by a general failure of interoception^28–31^. Alexithymia has been associated with poorer interoceptive accuracy skills^32–34^. Anxiety has been also associated with reduced interoceptive abilities^6, 35–38^, and interoceptive training seems to reduced anxiety scores in healthy volunteers^39^. Also, depression is usually characterized by interoceptive impairments and poor interoceptive accuracy^35, 38, 40, 41^. It is likely that both temporal and spatial locations of heartbeat sensations may be affected in these clinical and subclinical groups^6, 8, 23, 28, 35^, but such relationships have never been tested to date.

Similarly, individual differences in physical constitution, notably body mass index (BMI)^9, 10, 12, 13, 25, 42–44^ and cardiovascular parameters (e.g. interbeat intervals, heart rate, heart rate variability)^9, 42, 45^ may shape when and where heartbeat sensations are perceived^39^. On one hand, studies suggest that BMI is impacting heartbeat perception^25, 44^. On the other hand, there is some evidence to show no direct influence of body mass index^9, 12, 13, 42^, body weight, height or obesity index^10^ on heartbeat detection. However, body composition, as defined by body fat percentage, does have an impact: Leaner individuals tend to be better heartbeat perceivers than less lean people^43^. Moreover, a higher body mass index predicted a greater variety of spontaneous sensations felt in the hand, in interaction with heartbeat perception accuracy^25^. Given the mixed evidence in the literature, it seems important to test in this current study if the BMI is or not related to temporal and spatial heartbeat perception. Concerning cardiovascular parameters, some studies report that neither the length of the cardiac cycle^9^ nor resting heart rate^42^ was associated with differences in the temporal position (timing) of heartbeat perception. However, one study using a ‘multi-interval’ heartbeat discrimination task (in which heartbeats are judged for synchrony relative to auditory tones) showed that good heartbeat perceivers had slower heart rate and reduced heart rate variability than poor heartbeat perceivers^45^. To clarify the contribution of such factors to temporal and spatial heartbeat perception is crucial as it may be important biases to take in account in future studies using similar paradigms.

## Aims

The present study aimed to examine in detail the perception of heartbeat sensations, using a multi-interval heartbeat discrimination task^10^, to address the following questions:

- *When* do people perceive their own heartbeats ?
  We quantified the timing of heartbeat perception using a simultaneity judgement of heartbeat sensation relative to the presentation of an external auditory tone, triggered at different delays (SOAs) from the ECG R-wave. After listening to an individual sequence, the participant decided whether or not the tones were played simultaneously with perceived own heartbeats. Then, participants were asked to rate their confidence in these judgements. We predicted that participants would judge tones delivered between 100 and 300 ms after R-wave as simultaneous with their heartbeat^9, 10, 12, 16^.
- *Where* do people feel their heartbeat?
  On a two-dimensional body map, participants marked the anatomical location of maximal heartbeat sensation. We predicted that participants would typically feel their heartbeat in the left chest and neck areas^23, 24^.
- Does the timing of heartbeat perception relate to the spatial location of heartbeat perception?
  We tested if the observed timing of heartbeat sensation was predicted by the anatomical location and spatial dispersion of the heartbeat sensation.
- What factors influence individual differences in the temporal and spatial perception of heartbeats?
  Given the mixed evidence found in the literature, we tested for relationships between temporal and spatial differences in heartbeat perception and somatic (body mass index; BMI), and physiological measures (inter-beat interval, heart rate variability). We hypothesized that BMI would not have a marked impact on temporal or spatial aspects of heartbeat perception^9, 10, 12, 42^. Concerning physiological measures, we predicted that heart rate would impact heartbeat perception—namely, slower heart rate (i.e. longer inter-beat intervals) would be associated with better heartbeat perception^45^.
  Finally, we also explored how subclinical affective measures such as Anxiety, Depression, and Alexithymia related to temporal and spatial perception of heartbeats. We predicted that individual differences in affective symptoms will influence both aspects of heartbeat sensation^8, 23, 28, 35^.

## Results

**Figure 1:**
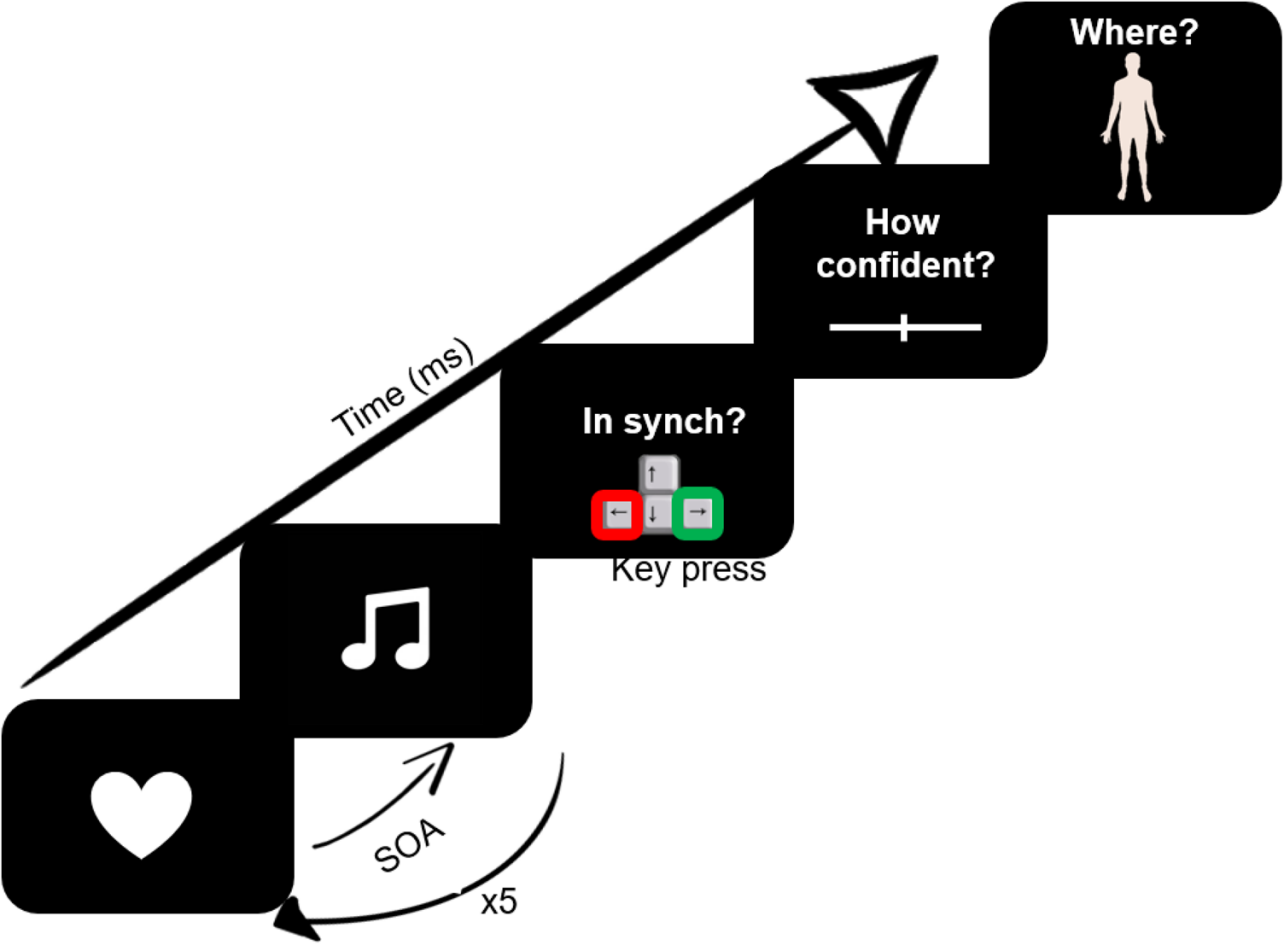
Schematic representation of a trial of the multi-interval heartbeat discrimination task. On each trial, the participant’s ECG R-peak was the priming trigger for tone presentations at a specific delay (0 to 500 ms), repeated 5 times per trial. The participant then judged the perceived simultaneity of the tones with their own heartbeats, rated confidence in that judgement, and marked where on the body the heartbeat sensation was felt. This figure has been created by Dr Sophie Betka.

Here, we examined the perception of heartbeat sensations, using a multi-interval heartbeat discrimination task (^9^; see Fig. 1 and the Methods section). On each trial, the participant listened to a sequence of 5 tones, which were all presented either 0, 100, 200, 300, 400 or 500 ms after the R-wave on ECG. After listening to an individual sequence, the participant decided whether or not the tones were played simultaneously with perceived own heartbeats. The participant then rated confidence in that decision, using a visual analogue scale (from 0 = not at all to 100 = completely). The participant was then presented with an outline image of a body on the screen and was asked to mark on the body template where did they feel their heartbeat sensation the strongest. Descriptive and Bayesian statistics alongside traditional mixed-effects linear models, contrasts and correlations were used to answer the following questions.

- When do people perceive their heartbeats in relation to an external auditory tone and how confident are they in that judgment?
- The temporal locations of heartbeat sensations happen around 250 ms after the ECG R-wave.

On average, participants felt their heartbeat 257.40 ms ± 31.31 ms (*Median* = 258.70 ± 55.77) after the actual ECG R-peak (see Table S1). Their modal preferred interval was 300 ms and ranged from 0 to 500 ms; its mean was equal to 265.40 ms. The specificity of discrimination (standard deviation of the modal preferred interval)^11^ was 149.36 ms. Individual performance is depicted in Figure S1.

**Figure 2:**
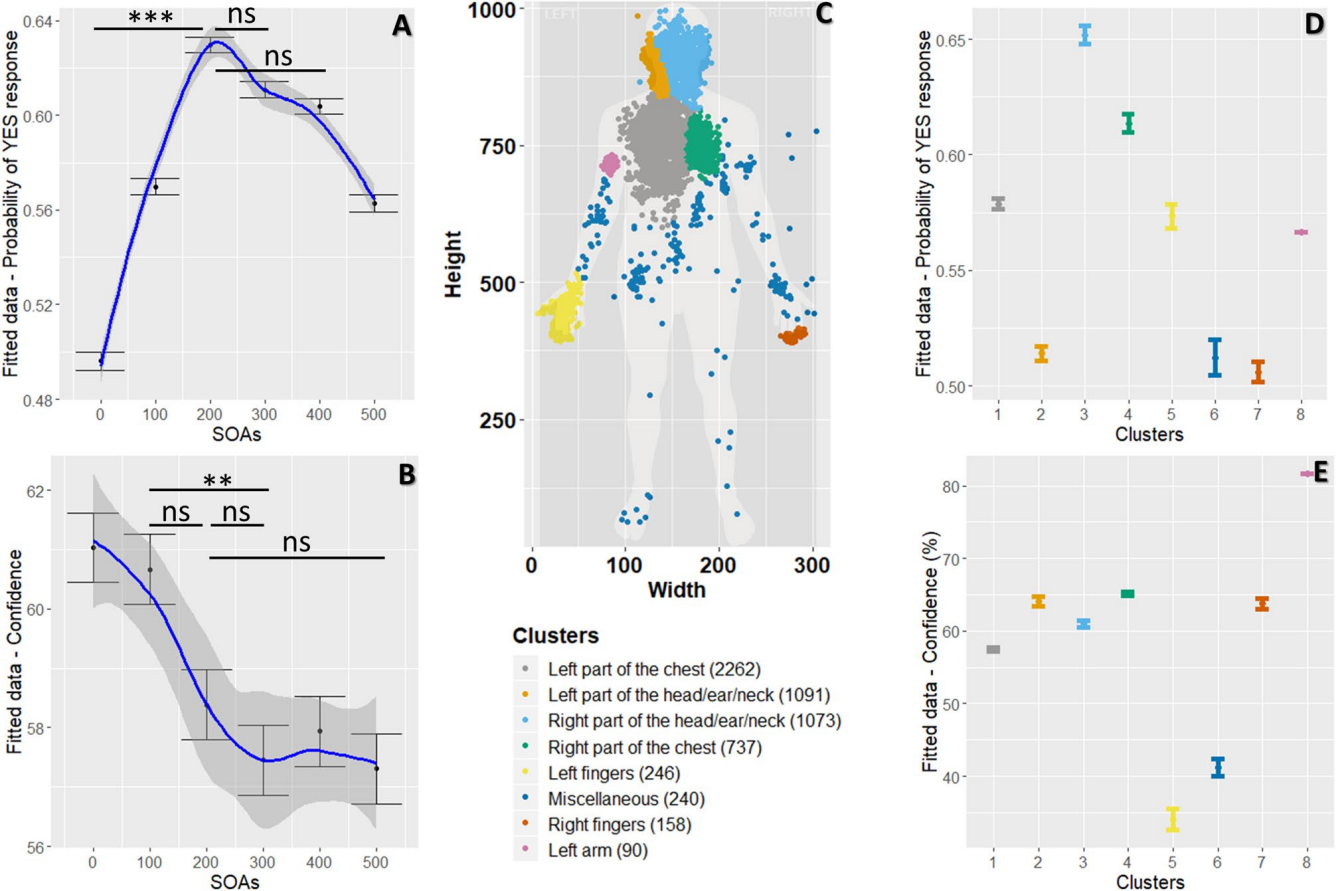
Results from the temporal and spatial heartbeat perception. (**A**) Temporal perception of heartbeat sensations: Graph of the effect of delays (stimulus onset asynchrony; SOAs) on the probability of yes response to simultaneity judgments of heartbeat with auditory tone (showing mean and error bars representing standard errors), while taking in account within-participant variability. (**B**) Confidence in the temporal perception of heartbeat sensations: Graph of the effects of delays (SOAs) on confidence ratings (with mean and error bars representing standard errors), while considering within-participants variability. (**C**) Visual representation of the eight clusters where participants reported sampling their heartbeat sensations. Each dot represents a trial. (1 = Left part of the chest, 2 = Left part of the head/ear/neck, 3 = Right part of the head/ear/neck, 4 = Right part of the chest, 5 = Left fingers, 6 = Miscellaneous, 7 = Right fingers, and 8 = Left arm).The number of observations is written in brackets for each cluster. This figure has been created by Dr Sophie Betka. (**D**) Graph of the effect of clusters on the probability of yes response (with mean and error bars representing standard errors), while considering within-participants variability. (**E**) Graph of the effect of clusters on confidence ratings (with mean and error bars representing standard errors), while considering within-participant variability. Significance of the main results are indicated.by ns = non-significant; **p values < .01; ***p values < .001*.*

Results of the mixed-effects regression model provided further insight (see Fig. 2A and Table S2) We report 95% confidence interval (95% CI), Savage-Dickey density ratio Bayes Factor (*BF*), the most credible value, and 95% Credible Interval (95% CrI): We observed that a delay of 200 ms from R-wave produced the highest probability of answering “Yes” for a judgment of simultaneity (β = 0.59, *SE* = 0.11, 95% CI [0.40, 0.76], *p* value < 0.001, *BF* = 252.44, 62.84%, 95% CrI [58.78, 66.99]), compared to the non-delay condition (β = − 0.007, *SE* = 0.141, 95% CI [− 0.20, 0.20], *p* value = 0.961, *BF* = 0.01, 49.66%, 95% CrI [45.62, 53.90]).

Based on previous work, we predicted that participants would judge tones delivered between 100 and 300 ms after R-wave as simultaneous with their heartbeat^9, 10, 12, 16^. Planned contrasts between delays from 100 to 300 ms were therefore computed. Interestingly, no difference in terms of probability of answering ”Yes” was observed for a delay of 100 ms compared to 200 ms (β = − 0.27, *SE* = 0.09, 95% CI [− 0.50, − 0.03], corrected *p* value < . 05 , *BF* = 0.3, 6.05%, 95% CrI [0.27, 11.84]), and compared to the 300 ms delay condition (β = − 0.18, *SE* = 0.09, 95% CI [− 0.41, 0.05], corrected *p* value = 0.157, *BF* = 0.04, − 4.25%, 95% CrI [− 9.84, 1.90]). No difference was observed between the 200 ms and the 300 ms delay conditions (β = 0.09, *SE* = 0.09, 95% CI [− 0.15, 0.32], corrected *p* value = 0.418, *BF* < 0.01, 1.89%, 95% CrI [− 3.99, 7.49]). A post-hoc contrast showed no significant difference in the probability of answering ”Yes” between the 200 ms and the 400 ms delay conditions (β = 0.12, *SE* = 0.093, 95% CI [− 0.06, 0.29], corrected *p* value = 0.418, *BF* = 0.01, 2.58%, 95% CrI [− 3.20, 8.47]). Our results demonstrate that participants felt their heartbeats between 100 and 400 ms (*Mean* = 257.40 ms).

The mean of confidence ratings (on 0–100 VAS) was equal to 58.78 ± 19.32 (*Median* = 62.59, *Rang*e = 3.53–97.6; see Table S1). Results of the mixed-effects regression model (see Fig. 2B and Table S4) revealed that lowest confidence ratings were observed for delays of 300 ms (β = − 3.65, *SE* = 0.91, 95% CI [− 5.43, − 1.87], *p* value = 0.001, *BF* > 1000, 57.47%, 95% CrI 55.67, 59.24]) and 500 ms (β = − 3.75, *SE* = 0.91, 95% CI [− 5.53, − 1.97], *p* value < 0.001, *BF* > 1000, 57.32%, 95% CrI [55.55, 59.11]), compared to the non-delay condition (β = 61.04, *SE* = 2.74, 95% CI [55.62, 66.46], *p* value < 0.001, *BF* > 1000, 60.97%, 95% CrI 59.20, 62.75]).

Based on published findings we predicted that confidence in timing simultaneity would relate to perceptual ease, and therefore be maximal for the 0 ms and 500 ms intervals, and minimal when discriminating simultaneity over the intervals between 100 and 300 ms. Planned contrasts were computed: A significant difference in confidence was observed between the 100 ms delay and the 300 ms delay condition (β = 3.24, *SE* = 0.091, 95% CI 0.97, 5.51], corrected *p* value < 0.01, *BF* = 23.25, 3.21%, 95% CrI [0.65, 5.73]). Moreover, no significant differences in confidence were observed between the 200 ms delay condition the 100 ms delay condition (β = 2.25, *SE* = 0.091, 95% CI [− 0.03, 4.52], corrected *p* value < 0.05, *BF* = 1.37, 2.26%, 95% CrI [− 0.27, 4.78]) , the 300 ms delay condition (β = 0.10, *SE* = 0.91, 95% CI [− 1.28, 3.27], corrected *p* value = 0.456, *BF* = 0.12, 0.94%, 95% CrI [− 1.57, 3.48]) or the 500 ms delay condition (β = − 0.1, *SE* = 0.091, 95% CI − 1.18, 3.37], corrected *p* value = 0.456, *BF* = 0.14, 1.08%, 95% CrI [− 1.45, 3.60]). Participants were thus more confident for the 0 ms interval, but less for intervals between 100 and 500 ms.

Not enough evidence was observed to validate or invalidate a relationship between mean temporal locations of heartbeat sensations and mean confidence (*r* = 0.212, *p* value = 0.13, *BF* = 0.87).

- Where do people feel their heartbeat?
- The spatial locations of heartbeat sensations happen in the left part of the chest.

After each trial, participants marked the anatomical site of maximal heartbeat sensation on a body map. The distance between the sampling location and the heart (assigned as a standardised location) was computed using coordinates marked by the participant on the body outline. Next, the mean of the distance to the heart was computed for each participant (Distance from the heart). We also computed dispersion from sampling locations by computing the mean of the standard deviation of X coordinates and the standard deviation of Y coordinates, for each participant ((sd(X) + sd(Y))/2). Finally, clusters of sampling location data points were defined using expectation–maximization algorithm for fitting mixture-of-Gaussian models (mclust R package, see methods section for details)^46^ and attributed to body parts and assigned names based on visual inspection. We isolated eight clusters (see Fig. 2C). The most frequently reported spatial location of heartbeat sensations (modal preferred cluster) was around the left part of the chest (cluster 1), consistent with previous work^23, 24^. Individual data are presented in Figure S2.

Results of the mixed-effects regression model are presented in Fig. 2D and Table S4. The highest number of simultaneity judgements were observed in fact for the right part of the head/ear/neck (β = 0.37, *SE* = 0.11, 95% CI [0.15, 0.59], *p* value = 0.001, *BF* = 720.06, 65.14%, 95% CrI [61.42, 68.87]) and the right part of the chest (β = 0.48, *SE* = 0.14, 95% CI [0.21, 0.77], *p* value = 0.001, *BF* = 273.05, 61.33%, 95% CrI [56.45, 66.08]) in contrast to the left part of the chest (β = 0.26, *SE* = 0.10, 95% CI [0.06, 0.46], *p* value = 0.01, *BF* = 0.26, 57.82%, 95% CrI [55.08, 60.61]). Counter-intuitively, these findings indicate that, when participants sample their heartbeat sensations from the right part of their head/ear/neck or chest, a greater probability of replying “Yes” was observed compared to when the participants sample their heartbeat sensations from their left part of the chest.

In terms of confidence, results of the mixed-effects regression model (Table Fig. 2E and Table S5) revealed that highest discriminatory confidence was observed for the left part of the head/ear/neck (β = 8.15, *SE* = 1.30, 95% CI [5.6, 10.70], *p* value < 0.001, *BF* > 1000, 64.06%, 95% CrI [62.34, 65.79]) compared to the left part of the chest (β = 55.75, *SE* = 2.52, 95% CI [50.78, 60.73], *p* value < 0.001, *BF* > 1000, 56%, 95% CrI [56.35, 58.78]).

- Does the timing of heartbeat perception relate to the spatial location of heartbeat perception?

**Figure 3:**
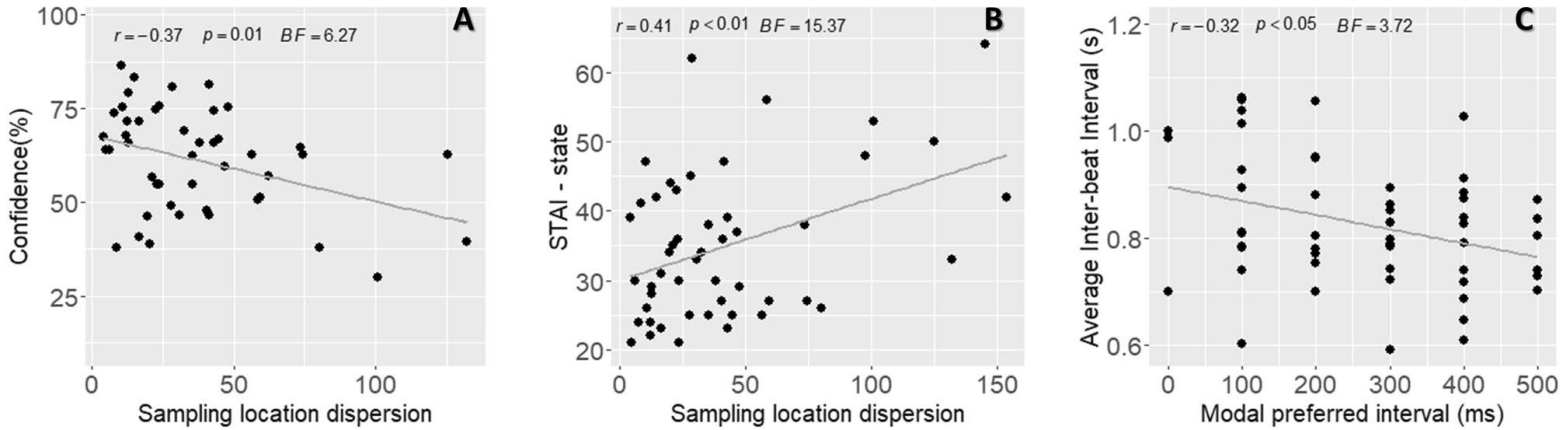
Correlations between heartbeat sensation parameters, interindividual differences and physiological measures. (**A**) Pearson correlation with coefficient *r, p* values, *BF* (Bayes Factor) for the relationship between confidence and sampling location dispersion. (**B**) Pearson correlation with coefficient *r, p* values, *BF* for the relationship between the STAI-S scores (state anxiety) and sampling location dispersion. (**C**) Pearson correlation with coefficient *r, p* values, *BF* for the relationship between the average interbeat intervals (IBI), and the modal preferred intervals.

We tested if the timing of, perceived heartbeat sensations and confidence in the simultaneity judgement were related to the distance from the heart of the indicated location of sampling, and/or the spatial dispersion of the sampling location (see Table 1). Pearson correlation coefficient, *p* value and Bayes factor (*BF*) were computed for each relationship. Higher confidence was associated with reduced sampling location dispersion (see Fig. 3A). However, evidence toward no relationship was observed between the standard deviation of heartbeat temporal perception and distance from the heart and also between the median of heartbeat temporal perception and the sampling location dispersion. The remainder of such relationships were characterized by a *BF* between 3 and 1/3 indicating that there was insufficient evidence in either direction to make a firm conclusion^47, 48^.

• What determines individual differences in the temporal and spatial perception of heartbeats?

**Table 1:** Pearson correlation coefficients *r*, *p* values and Bayes Factor (*BF*) for correlations between temporal (Average, standard deviation, median, mode, confidence) and spatial (Distance from the heart, sampling location dispersion) heartbeat sensation parameters. *BF* supporting evidence for a relationship (*BF* > 3) or supporting the absence of a relationship (*BF* < 1/3) between the variables are represented in bold.

	Distance from the heart			Sampling location dispersion		
	*r*	*p* value	*BF*	*r*	*p *value	*BF*
Average	0.151	0.296	0.521	− 0.074	0.611	0.358
Standard deviation	− 0.038	0.793	**0.328**	0.126	0.383	0.449
Median	0.176	0.222	0.623	− 0.033	0.82	**0.326**
Mode	0.177	0.218	0.632	− 0.047	0.744	0.334
Confidence	− 0.175	0.225	0.618	− 0.37	0.01	**6.265**

Perceptual accuracy on heartbeat detection tasks has been linked to a ‘slow and steady’ heart rate^45^ and thus may be diminished by increased heart rate variability (HRV and respiratory sinus arrhythmia, are usually associated with slower heart rate). HRV, perhaps also through accompanying changes in stoke volume, may also decrease confidence in, and add variability to the spatial precision of heartbeat perception. In contrast, heartbeat perceptual accuracy may be increased by sampling cardiac sensations from somatosensory locations that can offer greater sensory precision. Finally, individual differences in body mass and emotional state may directly or indirectly influence specific aspects of heartbeat perception. Therefore, we tested for correlations between temporal and spatial differences in heartbeat perception and participants’ somatic (BMI), psychological (Anxiety, Depression, Alexithymia) and physiological measures (inter-beat interval, heart rate variability) (see Tables 2, 3).

**Table 2:** Pearson correlation coefficients *r*, *p* values and Bayes Factor (*BF*) for correlations between heartbeat sensation temporal and spatial perception parameters, confidence, and psychometric/demographic parameters (BMI = body mass index; BDI = depression scores; STAI-S = anxiety state scores; STAI-T = anxiety trait scores; TAS = alexithymia scores). *BF* supporting evidence for a relationship (*BF* > 3) or supporting the absence of a relationship (*BF* < 1/3) between the variables are represented in bold.

	Average			Confidence			Distance from the heart			Sampling location dispersion		
	*r*	*p *value	*BF*	*r*	*p *value	*BF*	*r*	*p *value	*BF*	*r*	*p *value	*BF*
BMI	− 0.191	0.176	0.717	− 0.06	0.673	0.339	0.168	0.244	0.587	0.125	0.388	0.445
BDI	− 0.098	0.489	0.388	− 0.045	0.753	**0.327**	0.134	0.355	0.469	0.15	0.298	0.519
STAI-S	− 0.059	0.679	0.338	− 0.233	0.097	1.09	0.285	0.045	1.942	0.41	0.004	**15.366**
STAI-T	0.01	0.942	**0.313**	− 0.152	0.283	0.527	0.26	0.068	1.429	0.187	0.193	0.685
TAS	0.091	0.52	0.377	0.011	0.937	**0.314**	0.03	0.836	**0.325**	0.155	0.283	0.536

**Table 3:** Pearson correlation coefficients *r*, *p* values and Bayes Factor (*BF*) for correlations between average inter-beat interval (IBI), its variability (IBI sd, HRV), psychometric/demographic parameters (BMI = body mass index; BDI = depression scores; STAI-S = anxiety state scores; STAI-T = anxiety trait scores;.TAS = alexithymia scores)., temporal and spatial perception of heartbeat sensation and confidence. *BF* supporting evidence for a relationship (*BF* > 3) or supporting the absence of a relationship (*BF* < 1/3) between the variables are represented in bold.

	IBI			IBI sd (HRV)		
	*r*	*p *value	*BF*	*r*	*p *value	*BF*
BMI	0.007	0.963	**0.313**	0.13	0.357	0.46
BDI	− 0.15	0.289	0.52	0.052	0.714	**0.332**
STAI-S	− 0.135	0.341	0.352	0.102	0.47	0.39
STAI-T	− 0.073	0.607	0.471	0.099	0.484	0.396
TAS	− 0.175	0.216	0.626	0.034	0.812	**0.321**
Average	− 0.039	0.784	**0.323**	− 0.06	0.675	0.339
Standard deviation	− 0.153	0.28	0.53	0.046	0.743	**0.328**
Median	− 0.032	0.825	**0.32**	− 0.125	0.377	0.445
Confidence average	0.158	0.263	0.552	0.002	0.991	**0.313**
Mode	− 0.323	0.019	**3.718**	− 0.214	0.128	0.894
Distance from the heart	0.037	0.797	**0.328**	− 0.113	0.433	0.42
Sampling location dispersion	− 0.016	0.913	**0.32**	0.162	0.26	0.564
Preferred cluster	− 0.018	0.481	0.395	0.021	0.073	1.347

In this non-clinical sample, evidence supporting no relationship was observed between temporal location of heartbeat sensation and trait-anxiety scores (STAI-T), between confidence, depression and alexithymia scores (BDI and TAS) and between alexithymia scores and distance from the heart. State anxiety was associated with a more variable regional sampling of heartbeat sensations (see Fig. 3B). Interestingly, relationships between STAI-S and sampling location dispersion survived correction for mean inter-beat interval and HRV (Dispersion correcting for mean IBI *r* = 0.394, *p* < 0.01; and HRV: *r* = 0.385, *p* < 0.01). A lack of evidence in either direction to make a firm conclusion was observed for the remainder of the relationships.

We also observed that participants with slower heart rate preferred shorter time intervals for temporal location of heartbeat sensation (see Fig. 3C). However, evidence supporting no relationship was observed between interbeat-interval and BMI, heartbeat perception timing, its median, distance from the heart and sampling location dispersion (see Table 3). Concerning the heart rate variability HRV, evidence supporting the absence of a relationship was observed for depression scores, alexithymia, standard deviation of heartbeat perception timing, and confidence. A lack of evidence in either direction to make a firm conclusion was observed for the rest of the relationships.

To sum up, we found that heartbeat sensations occurred on average 250 ms after the ECG R-wave and were more frequently sampled from the left part of the chest. Individuals who felt heartbeats on the right of their upper body (head/ear/neck or chest) showed a greater probability of replying ‘Yes’ to heartbeat simultaneity judgments compared to those sampling from the left side of the chest. Participants’ confidence in their decision about simultaneity between heartbeat sensation and auditory tone presentation was maximal for the 0 ms and was lower after 100 ms the ECG R-wave. Interestingly, higher confidence was related to reduced dispersion of sampling locations. We found evidence supporting the absence of relationship between temporal and spatial heartbeat sensations perception. Finally, we found evidence toward a relationship between spatial precision of heartbeat sensations and state anxiety score, which seems independent from the cardiovascular parameters.

## Discussion

Altogether, we find that, on average, heartbeat perception seems to occur maximally 250 ms after the ECG R-wave and to be more frequently sampled from the left part of the chest. These findings, from our rigorous application of a multi-interval task comparing the inner heartbeat rhythm with an external rhythm of tones, extend evidence from previous studies concerning interoceptive processing, its integration with external stimuli, and conscious access to bodily signals^9, 12, 16, 23, 24^. Moreover, we observed that participants’ confidence in their experience of simultaneity judgement—between tones and their heartbeat sensations—was maximal for the 0 ms intervals and was lower after 100 ms the ECG R-wave. Even though the left part of the chest was the most frequent location of heartbeat sensation, those individuals who felt heartbeats on the right of their upper body (head/ear/neck or chest) showed a greater probability of replying ‘Yes’ to heartbeat simultaneity judgments compared to those sampling from the left side of the chest. Speculatively, the observed pattern of perceptual lateralization may have a basis in peripheral (left versus right vagus nerve) anatomy (Craig 2002) and central neural organisation where interoceptive inputs, integrated within right and the left anterior insula respectively, are putatively re-represented in the dominant right anterior insula^3^. Arguably, this may also mean that, in general, most participants base their judgments of cardiac timing and synchrony on spinothalamocortical information rather than vagus nerve afferents. However, such right-side dominance merits further evaluation, not least because it was not reflected in confidence ratings for the simultaneity judgments; higher judgment confidence was signalled for heartbeat sensations felt in the left head/ear/neck compared to left chest. Nevertheless, there remains some coherence with the hypothesis of right cerebral hemisphere engagement in the representation of heartbeat sensations attributable to peripheral cardiovascular asymmetries^20, 49, 50^. Confidence in heartbeat sensations may also be affected by pre-existing beliefs and biases arising from the participants’ understanding of anatomy (including knowledge that the heart is placed largely in the left part of the chest) and by quasi-interoceptive somatosensory pathways from the chest wall or the skin (see^51^) , which may afford great perceptual precision relative to viscerosensation. We have some evidence to support this second explanation, notably that higher confidence was associated with reduced dispersion of the sampling location; thus, better spatial precision that may suggest a greater somatosensory contribution. Indeed, it has been shown that the somatosensory (exteroceptive) pathway that gathers information from the skin and from Pacinian and non-Pacinian somatosensory mechanoreceptors located on the chest wall also contributes to the spatial precision of heartbeat perception^21, 51^. For example, when participants are asked to focus on heartbeats, not only the insula but also the somatosensory cortices are activated^20, 52^. Moreover, such cortical areas are proposed to be one of the sources of the heartbeat-evoked potential; a cortical signature of the heartbeat^53, 54^. Furthermore, interoceptive sensations, including visceral pain, are poorly localized and may be felt at sites distal to the affected organ, due to overlap between, recruitment of and misattribution of somatic versus visceral afferent information at different levels of the neuraxis^55–57^. In animals, some spinal afferent neurons can be activated by mechanical stimulations of the somatic receptive fields in the segments related to the head, jaw, neck and shoulder^5^. This may explain why in patients, shoulder pain associated with diaphragmatic involvement and left arm ache associated with cardiac angina^58–60^. Other evidence supporting poor spatial localization of visceral sensations lay in the fact that visceral organs are much less innervated by spinal afferents than the—superficial and deep—somatic tissues^61^. For example, in the cat, 22 to 25,000 spinal afferents project to the viscera, on a total of about 1 to1.5 million spinal afferent neurons. Also, while 30,000 to 40,000 afferent neurons project through the abdominal vagal nerve, the number of vagal afferent neurons innervating thoracic visceral organs is unknown^5^. Further neuroimaging work should investigate the relationship between heartbeat-related insular as well as somatosensory activation and the individualised spatial precision of heartbeat sensations. We would predict that a greater contribution of the somatosensory pathway (recruitment of spinal afferent neurons leading to greater somatosensory activations) will be associated with reduced sampling locations, but this needs to be explicitly tested.

A second question that we aimed to address was whether temporal and spatial perception of heartbeat sensation relate to each other. Simplistically, one might predict that a greater sampling distance of heartbeat/pulse perception from the heart would be associated with a greater lag in the perception of its timing, reflecting the inherent delay in the blood pulse wave’s activation of somatic mechanoreceptors and subsequent signaling to somatosensory/interoceptive cortices. In our present study, we found evidence against a simple relationship between timing of heartbeat perception with both the distance from the heart and the dispersion of sampling location. In fact, our findings suggest the absence of any clear consistent relationship between the temporal and spatial perception of heartbeats across individuals. Such results are in line with some previous exploratory findings suggesting no clear relationship between temporal and spatial aspects of heartbeat perception^10, 12^. One potential explanation of the absence of a relationship could be variance related different types of afferent fibres conveying the heartbeat-related information that need to be taken into account alongside the sampling localization site. Indeed, conduction velocities differ markedly between small-diameter interoceptive (C: 0.5–2 m/s; Aδ: 3–15 m/s) and large-diameter touch-specific (Aβ: 33–75 m/s; conveying information related to mechanical pressure or distortion of the skin) afferent fibres^62^. It is thus possible to sample heartbeat sensations from a same site, but, depending on the type of fibres activated (possibly also reflecting the strength of heartbeat), variations in timing of heartbeat perception might be observed. Also, it is postulated that many fibres—which are excited from the skin—ascending in spinal lamina I, may also receive convergent synaptic activation from small-diameter afferent neurons innervating deep somatic tissues (such as skeletal muscle, joint and/or viscera)^55, 63–65^. A second potential explanation of such absence of relationship could be that even though participants think they sample their heartbeat sensations from peripheral areas, more salient and central interoceptive afferents signals (e.g. from the activation of the baroreceptors) overwrite focal signals from the sampling location—or vice versa. Future high-field fMRI studies with higher spatial resolution could be leveraged to dissect the links between precise heartbeat-related somatosensory activations, related body parts representations and temporal/spatial heartbeat perception.

Our study further enabled us to test how individual characteristics contribute to heartbeat sensations. Physiologically, we showed that a slower heart rate was associated with a smaller preferred interval, but did not predict a specific timing of the actual heartbeat perception within the cardiac cycle (e.g. mean or median of intervals considered as in synch). This is interesting as both heart rate and HRV have previously been shown to influence performance in a multi-interval task, an effect proposed to arise because either people with slow heart rates have additional time to process cardiac sensations, or show differences in expectancies^45^. Given the absence of a relationship between heart rate and the temporal precision of heartbeat perception, our results rather support the notion that heart rate expectancies exert a potentially greater impact on performance. Another important, and to a degree unexpected, finding within this study of non-clinical individuals was the absence of a relationship between trait anxiety and the timing of heartbeat sensations. Indeed, disrupted interoceptive ability is widely described in people suffering from anxiety disorders^6, 23, 36, 37, 66^ relationships between interoceptive accuracy and anxiety score are frequently described in ‘analogue’ populations (e.g. see^67^). Nevertheless, our results suggest that specific cardiac-timing paradigms can be implemented effectively in sub-clinical anxious populations since non-clinical anxiety does not seem to modulate the temporal perception of heartbeat sensations. Nevertheless, we observed that higher levels of state anxiety were associated with lower spatial precision (increased sampling locations dispersion) even after controlling for cardiovascular parameters. Speculatively, people who feel their heartbeats reliably in the same anatomical location most likely are drawing upon somatosensory feedback (e.g. from the skin) rather than the less precise interoceptive feedback from viscerosensory afferents^21^. Our data suggest that state anxiety symptoms do not depend greatly on this somatosensory contribution to interoceptive experience. Finally, we tested whether BMI had a potentially explanatory or confounding effect on interoceptive measurements. However, interoceptive abilities might appear to be more influenced by the body fat or muscle percentages, and not by the BMI itself. Further studies should take in account this aspect.

Our results of this study should be considered in light of several constraints. First, headphones were used to deliver sounds to participants. Both ears are shown to be key areas of heartbeat sensation; areas highlighted in limited previous research^21–24^. This may suggest that the pressure of the headphones may have given somatosensory feedback and influenced the location of heartbeat sensation experienced by participants^68^. A second limitation was inherent to the chosen experimental paradigm. Indeed, one could think that focusing on both an external and internal signal is attentionally demanding and increases cognitive load at the expense of performance accuracy, especially with respect to our last question about the perceived spatial location of heartbeats). Further tasks—purely investigating interoceptive signals—should be developed to avoid such potential cofound. As people may report that they feel heartbeats where the clothing is too tight, further studies should include extra requirements regarding the clothing. Also, as shown by our use of Bayesian statistics (Bayes factors), larger participant samples are required to test the relationship between interindividual affective characteristics and heartbeat sensations to generate firm conclusions. Nevertheless, our investigation of the temporal and spatial perception of heartbeat sensations provides important, fresh insights for the fields of experimental psychology, psychiatry and neuroscience. These insights may inform studies involving neuroimaging, ideally to disentangle the contributions from both interoceptive and somatosensory neural pathways, and will help build upon a mechanistic understanding of embodiment, individual differences, and the contribution of interoceptive signaling to emotion, cognition and behaviour.

## Methods

### Participants

Sixty-two volunteers (29 males, 33 females) aged from 18 to 45 years (*M* = 23.48 years, *SD* = 4.69) were recruited via advertisements at the University of Sussex and Brighton and Sussex Medical School. Given a medium effect size (eta partial square = 0.27 se^12^), an alpha of 0.05, a beta of 0.85, a minimum of 48 participants needed to be recruited. We recruited more than 48 participants in anticipation of potential outliers.

All participants were healthy individuals with no history of psychiatric or neurological diseases and were not taking medication. One participant did not meet the inclusion criteria and was excluded from the study—the reason for the exclusion of the other participants will be elaborated later and described in the Supplementary section. Participants were informed that they would complete a series of psychometric questionnaires and would take part in two tasks for one and a half hour. All participants gave their written informed consent and were compensated for their time (£15). The study was reviewed and approved by the BSMS Research Governance and Ethics committee. All methods were performed in accordance with the relevant guidelines and regulations.

### Demographic and psychometric description of the sample

The final sample was composed of 52 participants (26 Females, age: *M* = 22, *Median* = 22, *SD* = 4.7, *Range* = 18—45, years of education: *M* = 16.41, *Median* = 16, *SD* = 2.4, *Range* = 11–21). Characteristics of the sample and psychometric measures are presented in Table 4.

**Table 4:** Demographic and Psychometric measures: Minimum, 1st quartile, Median, Mean, standard deviation, 3rd quartile and maximum of Body Mass Index (BMI) and psychometric measures.

	BMI	BDI	STAI-S	STAI-T	TAS
Min	16.61	0.00	20.00	20.00	26.00
1st Qu	20.31	2.00	25.75	32.00	41.75
Median	22.15	4.50	32.00	38.00	47.50
Mean	22.21	7.94	34.25	41.44	48.56
Sd	2.70	9.34	10.84	11.77	11.20
3rd Qu	23.73	9.50	41.25	50.25	56.00
Max	30.78	43.00	64.00	65.00	72.00

### Procedure

The study was conducted in dedicated human testing facilities at the University of Sussex. Participants gave demographic information (e.g. age, weight, height) and a set of completed questionnaires, before the experiment. They next performed an audio-visual simultaneity task (for familiarisation with task demands) followed by the multi-interval heartbeat discrimination task that shared the same design structure.

### Questionnaires

#### Beck depression inventory (BDI)

The presence of depressive symptoms in participants was quantified using the Beck Depression Inventory (BDI). This questionnaire consists of 21 questions measuring cognitive, affective and somatic symptoms of depression as experienced by participants in the last 2 weeks. Each question is scored 0–3 with a higher number indicating a greater degree of symptom severity; allowing for a total score of up to 63. A score of 14–19 suggests mild depression, 20–28 suggests moderate depression and 29–63 indicates severe depression^69^. In the current sample, the Cronbach's α was equal to 0.927 ( 95% CI [0.859, 0.955]).

#### Toronto alexithymia scale-20 items (TAS-20)

The TAS-20 consists of 20 items rated on a five-point Likert scale (from 1 ‘strongly disagree’ to 5 ‘strongly agree’). The TAS-20 is composed of three factors (F1, F2, F3). The first-factor measures difficulties in identifying feelings (DIF), the second factor measures difficulties in describing feelings (DDF) and the third-factor measures the way the participant uses externally oriented thoughts (EOF). The total alexithymia score is the sum of responses across all 20 items. We considered the total score only in our analyses. A score equal or inferior to 51 suggests no alexithymia, a score equal or superior to 61 suggests alexithymia and a score between 52 and 60 suggests possible alexithymia^70^. In the current sample, the Cronbach's α was equal to 0.814 ( 95% CI [0.728, 0.864]).

*Anxiety Inventory* (STAI-T & S) Trait anxiety was assessed using the Trait version of the Spielberger State/Trait Anxiety Inventory (STAI-T). This questionnaire is composed of 20 questions, assessing trait anxiety with questions such as “I lack self-confidence” and “I have disturbing thoughts”. Participants were asked to answer each statement using a response scale that runs from ‘Almost never’ to ‘Almost always’ to establish if there was a stable dispositional tendency (trait) for anxiety^71^. In the current sample, the Cronbach's α was equal to 0.939 (95% CI [0.914, 0.956]). State anxiety was assessed using the State version of the Spielberger State/Trait Anxiety Inventory (STAI-S). This questionnaire is composed of 20 questions, assessing trait anxiety with questions such as “I feel calm” and “I feel worried”. Participants were asked to answer each statement using a response scale that runs from ‘Not at all’ to ‘Very much so’ to establish if there was a transitory emotional reaction (state) of anxiety. A cutoff score of 40 is commonly used to define probable clinical levels of anxiety^71^. In the current sample, the Cronbach's α was equal to 0.931 ( 95% CI [0.889, 0.954]).

### Apparatus and task

Participants were comfortably sat on a desk chair and viewed a 24-inch monitor at an approximate distance of 50 cm. The monitor’s visual display had a screen resolution of 1920 × 1200 pixels and a refresh rate of 60 Hz. Auditory stimuli were delivered to participants through headphones. Experimental task procedures were implemented as in-house programmes using Psychophysics Toolbox Version 3 (http://psychtoolbox.org/) running in Matlab R2013a (The MathWorks, Inc., Natick, MA; https://ch.mathworks.com/products/matlab.html).

During the Multi-interval heartbeat discrimination task, tones were synchronised to specific time points within the cardiac cycle using electrocardiography (ECG) implemented via Cambridge Electronic Design (CED) hardware and Spike2 physiological recording software (version 7.18; http://ced.co.uk/products/spkovin). Cardiac events were interfaced with the task events in Matlab. Three Ag/AgCl electrodes (3 M Healthcare, Neuss, Germany) were attached with foam tape: two on the upper left and right chest, and a ground electrode above the left hipbone. The ECG signal was sampled at 1000 Hz, amplified (1902, CED) and relayed to Spike2 recording software via an analogue-to-digital recorder (1401, CED). An inter-active threshold in the Spike2 recording isolated each ECG R-wave peak, which then primed tones delivery in the Matlab task script. The computer was equipped with a Strix Soar (https://www.asus.com/us/) soundcard allowing an input–output latency < 10 ms and SNr > 110 dB. The mean of the input/output latency was equal to 6.78 ms and its standard error was equal to 0.95 ms.

In the multi-interval heartbeat discrimination task, each participant was required to judge the simultaneity of a sequence of 5 tones with his/her own heartbeat (see Fig. 1)^9, 11^. This computerised task examines the participant’s ability to integrate interoceptive (heartbeat sensation) and exteroceptive (auditory stimuli) signals. Participants were comfortably sat on a desk chair with both arms on the desk, and one hand on the mouse. Before the beginning of the task, the participant was instructed not to palpate his/her own pulse at any point during the experiment. Sequences of 5 tones were played to the participant, primed by his/her own ECG R-peaks. The tones of the sequence were delayed by one of the six time-intervals (SOA: 0, 100, 200, 300, 400, 500 ms) after the ECG R-peak. The participant had to decide whether the tones were played simultaneously with his/her own heartbeat or not and, then, rate how confident was that decision, using a visual analogue scale (VAS) on a computer screen. The participant was then presented with an outline image of a body on the screen and had to click on the body where the heartbeat sensation was felt the strongest. Coordinates (x,y) of the selected body part were recorded. Each tone was played for a duration of 0.5 s at a frequency of 48000 Hz. The time interval between each question was equal to 1 s and inter-trial interval (i.e. period between the trials) was equal to 5 s. The time-interval between each tone of the sequence was driven by the actual heartbeats of the participant. Overall, 120 trials were presented in a randomized order, with 20 trials per interval. These trials were completed over four separate 30-trial blocks with opportunities for rest in between blocks if required. The task lasted approximately 45 min. For two participants, we failed to record the location of heartbeat sensations. The familiarisation task (an audio-visual simultaneity task with the same design structure) is described in the supplementary section.

### Data analyses

Data were checked for outliers. One participant did not perform the audio-visual simultaneity task correctly (i.e. replied yes for all trials), three participants were not able to perform multi-interval heartbeat discrimination task (i.e. probability did not reach 0.50 of ‘yes’ response for any SOAs). These participants were removed from subsequent analyses. After computing the mean confidence and just-noticeable difference (described in the Supplementary section) for the audio-visual simultaneity task, six participants whose performance data fell outside 1.5 times the interquartile range above the upper quartile and below the lower quartile were labelled as outliers and their data was excluded^72^. The final sample size was equal to N = 52 (including 2 participants without localization data due to technical issues).

Each participant self-reported their height and weight. The body Mass Index (BMI; weight (kg) / height (m)^2^) was computed for each participant. All demographic, psychometric, and performance data for both tasks were held in long format (for mixed-effects effects models analyses) and short format in an averaged form (for correlations and descriptive statistics).

For each participant, the average interval (of reported heartbeat synchrony), its standard deviation, median and mode were calculated. The mode was used to assess the preferred time interval as being “in sync” with the heartbeat (i.e. temporal location of heartbeat sensation). The mean confidence and mean inter-beat interval (IBI) duration during the task were also computed. The standard deviation of the inter-beat interval (IBI SD) duration during the task was calculated and used as a marker of heart rate variability (HRV).

Concerning spatial perception of heartbeat sensation, for each trial, the distance between the sampling location and the heart (assigned a standardised location) was computed using coordinates marked by the participant on the body outline. Next, the mean of the distance to the heart was computed for each participant (Distance from the heart). We also computed dispersion from sampling locations by computing the mean of the standard deviation of X coordinates and the standard deviation of Y coordinates, for each participant ((sd(X) + sd(Y))/2). Finally, clusters of sampling location data points were defined using expectation–maximization (EM) algorithm for fitting mixture-of-Gaussian models (mclust R package, version 5.4.7, https://cran.r-project.org/web/packages/mclust/index.html)46 and attributed to body parts and assigned names based on visual inspection.

### Statistical analyses

All analyses were conducted in the R environment^73^ (version 3.6.1; https://www.r-project.org/). Descriptive statistics were computed for all variables.

A non‐significant *p* value is not enough to provide evidence toward the null hypothesis or toward the fact that the data are insensitive and that additional data are needed to conclude^74, 75^. Therefore, to facilitate the interpretation of our data, we ran separate Bayesian analyses, computing the Bayes Factor (BF) to indicate the strength of evidence; *p* values were used as the basis of decision making in respect of the compared hypotheses. Differences were considered significant when the probability *p* of a type I error was below 0.05.

Linear mixed-effects models were used in the analysis of confidence measures as the outcome was continuous. Generalized linear mixed models were used to analyse simultaneity assessment probability as the outcome was binary (non-simultaneous = 0; simultaneous = 1; binomial family function). In all models, participants were treated as a random factor with random intercepts^76^. For frequentist analyses, the lme4 package (version 1.1-26, https://cran.r-project.org/web/packages/lme4/index.html) was used^77^ and *p* values were computed using lmerTest package (version 1.1-26, https://cran.r-project.org/web/packages/lme4/index.html)77. 95% confidence intervals were computed and presented in each table. Two-sided contrasts were computed using the emmeans package (version 3.1-3, https://cran.r-project.org/web/packages/lmerTest/index.html)^79^ and *p* values were corrected following the Holm–Bonferroni method. Based on previous work, we expected that participants would judge tones delivered between 100 and 300 ms after initiation of ventricular contraction as simultaneous with their heartbeat^9, 10, 12, 16^. Planned contrasts between delays from 100 to 300 ms were thus computed. We predicted that confidence in timing simultaneity would relate to perceptual ease, and therefore be maximal for the 0 ms and 500 ms intervals, and minimal when discriminating simultaneity over the intervals between 100 and 300 ms. Planned contrasts were computed.

Bayesian models were created in Stan computational framework (http://mc-stan.org/) accessed with the brms package (version 2.14.4, https://cran.r-project.org/web/packages/brms/index.html)^80^. To improve convergence and guard against overfitting, we specified weakly informative conservative priors (normal(0, 10)). Iterations were set to 2000 and chains to 4, where iteration numbers could be increased to achieve convergence. For each model and two-sided contrasts, Bayes Factor (BF) against the null, based on prior and posterior samples of a single parameter was estimated using the bayestestR package (version 0.8.2, https://cran.r-project.org/web/packages/bayestestR/index.html)^81^. For contrasts, we also computed the most credible value and the 95% credible intervals (95% CrI in brackets), using brms package.

Two-sided frequentist Pearson correlations and partial correlations coefficients were calculated using Hmisc package (version 4.4-2, https://cran.r-project.org/web/packages/Hmisc/index.html)^82^ and Bayesian correlations, using BayesFactor package (version 0.9.12-4.2, https://richarddmorey.github.io/BayesFactor/).

A BF greater than 3 can be considered as substantial evidence against the null model, while a BF smaller than 1/3 indicates substantial evidence in favour of the null model^47, 48, 83^.

Our interpretations required coherence between *p* values and *BF*s (e.g. evidence for an effect was characterized by *p* value < 0.05 and *BF* < 3).

## Supplementary Information

Supplementary Information.

## Data Availability

The dataset and MATLAB scripts used in this study will be made available upon request by the corresponding author, Dr Sophie Betka (sophie.betka@epfl.ch). **Copyright** Figure 1, Fig. 2C and Figure S1 have been drawn by Dr Sophie Betka.
