# Supplementary material for: Evidence toward the potential absence of relationship between temporal and spatial heartbeats perception
Source: Sci Rep. 2021 May 24;11:10759. doi: 10.1038/s41598-021-90334-z (PMC8144555; doi:10.1038/s41598-021-90334-z)
Supplement: Supplementary file 1 — Supplementary Information. [file 41598_2021_90334_MOESM1_ESM.docx]

**Evidence toward the potential absence of relationship between temporal and spatial heartbeats perception**

Betka Sophie* ^1,2,3^, Łukowska Marta^4^, Silva Marta^1^, King Joshua^1^, Garfinkel Sarah^1,5^, Critchley Hugo^1,5^

1. Clinical Imaging Science Centre, Brighton and Sussex Medical School, Sussex, Brighton BN1 9RY, United Kingdom

2. Laboratory of Cognitive Neuroscience, Brain Mind Institute and Center for Neuroprosthetics, Faculty of Life Sciences, Swiss Federal Institute of Technology, (EPFL), Geneva, 1202, Switzerland

3. Department of Clinical Neuroscience, Faculty of Medicine, University of Geneva, Geneva, 1211, Switzerland

4. Consciousness Lab, Institute of Psychology, Jagiellonian University

5 Sackler Centre for Consciousness Science, University of Sussex, Sussex, BN1 9QJ, United Kingdom

# Supplementary section

## Removed outliers

| **Subject ID** | **Reason of the removal** |
| --- | --- |
| **01** | Probability did not reach 0.50 of ‘yes’ response for any SOAs on the heartbeat discrimination multi-interval task |
| **11** | Just-noticeable difference fell outside 1.5 times the interquartile range above the upper quartile and below the lower quartile on the audio-visual simultaneity task |
| **21** | Confidence fell outside 1.5 times the interquartile range above the upper quartile and below the lower quartile on the audio-visual simultaneity task |
| **22** | Was taking antidepressants (citalopram 30mg) |
| **24** | Just-noticeable difference fell outside 1.5 times the interquartile range above the upper quartile and below the lower quartile on the audio-visual simultaneity task |
| **37** | Probability did not reach 0.50 of ‘yes’ response for any SOAs on the heartbeat discrimination multi-interval task |
| **40** | Confidence fell outside 1.5 times the interquartile range above the upper quartile and below the lower quartile on the audio-visual simultaneity task |
| **43** | Just-noticeable difference fell outside 1.5 times the interquartile range above the upper quartile and below the lower quartile on the audio-visual simultaneity task |
| **46** | Probability did not reach 0.50 of ‘yes’ response for any SOAs on the heartbeat discrimination multi-interval task |
| **50** | Replied “yes” to for all trials of the audio-visual simultaneity task |

Table S1 Outliers

We did not manage to record heartbeats location data for subjects 33 and 54, due to technical issues. Such subjects were only discarded from heartbeats location data analyses.

## Audio-visual simultaneity task

### Methods


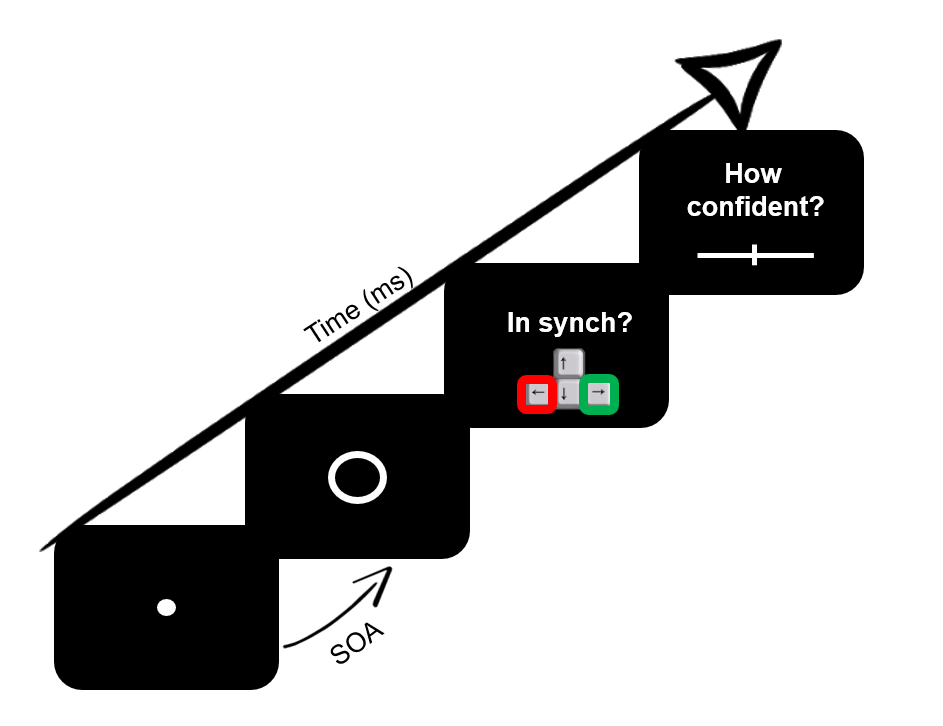


Figure S1 Schematic representation of a trial of the audio-visual simultaneity task. Stimulus-onset-asynchrony (SOA) = 0, 100, 200, 300, 400 and 500ms

In the audio-visual simultaneity task, subjects were required to judge the simultaneity of tones produced through their headphones with the appearance of a white circle on the screen (see Figure S1) (Brener et al., 1993; Brener & Ring, 2016). This task examines participants’ ability to integrate two different exteroceptive signals (auditory and visual stimuli). A small white dot appeared in the middle of the screen. This dot was quickly replaced by a white circle and a tone was produced through the subject’s headphones randomly at one of six pre-determined intervals (Stimulus-onset-asynchrony (SOA): 0, 100, 200, 300, 400 and 500ms) after the appearance of the white circle. At this point, the subject had to decide whether the circle and tone were produced simultaneously or not by pressing a specific key. After each trial, subjects rated how certain they were in their decision, using a confidence visual analogue scale (VAS; “0” – “I am guessing” to “100” – “I am sure”). Inter-stimulus interval was equal to 1s and inter-trial interval was equal to 5s. Overall, 90 trials with 15 trials per interval were presented. These trials were completed in 3 separate 30-trial blocks with opportunities for breaks in between each block. On average, this task took 15 minutes to complete.

By computing the number of yes response for each SOA, for each participant, we computed the point of subjective equality (PSE; that is the stimulus SOA that elicits 50% of yes responses) and the just noticeable difference (JND; the amount of change necessary to cause observers to perceive a difference in a signal). Mean confidence was also calculated.

Based on such performance, one participant (Subject 50) did not perform the audio-visual simultaneity task correctly (i.e. replied yes for all trials). After computing the mean confidence and just-noticeable difference participants whose performance data fell outside 1.5 times the interquartile range above the upper quartile and below the lower quartile were labelled as outliers and their data was excluded. (JND: Subjects 11, 24, 43; Confidence: Subjects 21, 40).

### Results

After removing the outliers, the sample was equal to 55. The average point of subjective equality (PSE) and just-noticeable difference (JND) were 228.71ms (*SD* = 76.68, *Median* = 229.29, *Range* = 84.46 – 419.81) and -76.64ms (*SD* = 28.85, *Median* = -73.40, *Range* = -145.69- 30.82), respectively, see Figure S2.

Individual performances are depicted on Figure S5.


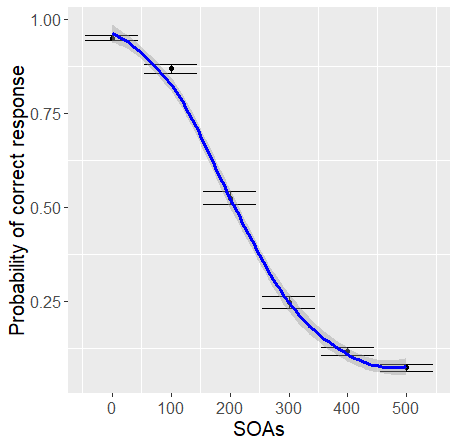


Figure S2 Audio-visual simultaneity task: Graph of the effects of delays (SOAs) on the probability of yes/correct response (with mean and error bars representing standard errors).

Mean confidence was 80.98 (*SD* = 9.05, *Median* = 81.34, *Range* = 60.13 – 96.69, see Figure S3).


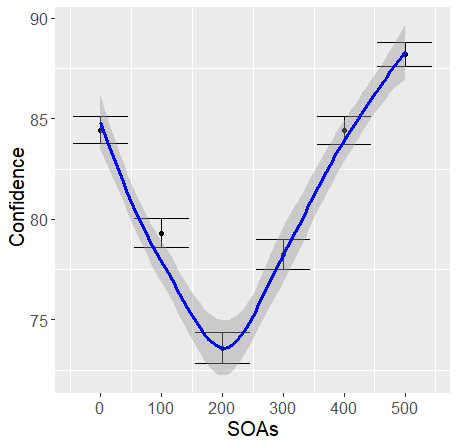


Figure S3 Audio-visual simultaneity task: Graph of the effects of delays (SOAs) on confidence (with mean and error bars representing standard errors).

### Correlations with demographic and psychometric measures

Correlations were computed (see Figure S4 & Table S2).

Greater confidence was associated with smaller PSE and smaller absolute value of JND.

Greater difficulty in identifying feeling was associated with smaller JND.

|  | PSE | | | JND | | |
| --- | --- | --- | --- | --- | --- | --- |
|  | *r* | *p-*value | *BF* | *r* | *p-*value | *BF* |
| BMI | -0.121 | 0.393 | 0.435 | -0.038 | 0.789 | **0.322** |
| BDI | 0.011 | 0.939 | **0.313** | 0.067 | 0.635 | 0.346 |
| STAI1 | 0.008 | 0.953 | **0.313** | 0.099 | 0.483 | 0.391 |
| STAI2 | 0.063 | 0.658 | 0.342 | 0.106 | 0.454 | 0.403 |
| TAS | -0.084 | 0.552 | 0.367 | 0.259 | 0.064 | 1.480 |
| Confidence | -0.294 | **0.034** | 2.385 | 0.387 | **0.005** | **11.931** |

Table S2 Pearson correlation coefficients *r*, *p*-values and Bayes Factor (*BF*) for correlations between Point of Subjective Equality (PSE), the Just Noticeable Difference (JND) and psychometric/demographic parameters.


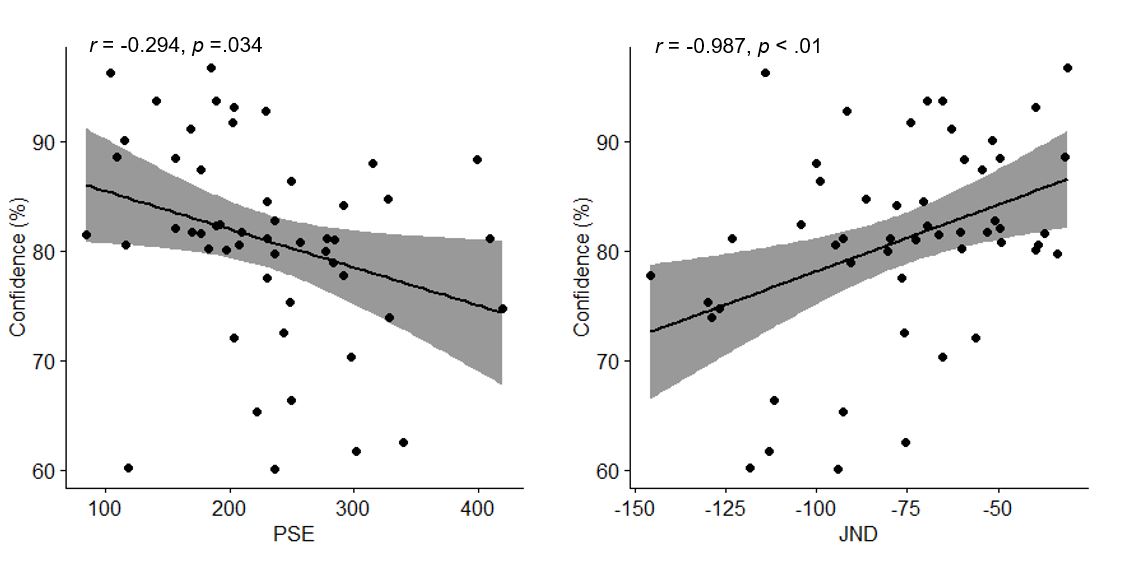


Figure S4 Pearson correlations with coefficients *r* and *p*-values for significant relationships between confidence and PSE and JND.

## When do people perceive their heartbeats in relation to an external auditory tone and how confident are they?

|  | Average | Standard Deviation | Median | Mode | Confidence | IBI (s) | Distance from the heart | Sampling location dispersion |
| --- | --- | --- | --- | --- | --- | --- | --- | --- |
| Min | 196.00 | 122.30 | 200.00 | 0.00 | 3.53 | 0.59 | 20.22 | 4.35 |
| 1st Qu. | 241.60 | 161.80 | 200.00 | 100.00 | 48.22 | 0.74 | 59.92 | 16.79 |
| Median | 254.00 | 166.60 | 300.00 | 300.00 | 62.59 | 0.81 | 91.70 | 31.64 |
| Mean | 257.40 | 163.30 | 258.70 | 265.40 | 58.78 | 0.83 | 111.52 | 42.69 |
| Std | 31.31 | 13.56 | 55.77 | 149.36 | 19.32 | 0.12 | 71.93 | 36.95 |
| 3rd Qu | 268.70 | 171.70 | 300.00 | 400.0 | 72.11 | 0.89 | 150.96 | 54.33 |
| Max. | 353.70 | 180.00 | 400.00 | 500.0 | 97.60 | 1.06 | 373.63 | 153.72 |

**Table S3 Temporal perception of heartbeat sensations**: Minimum, 1^st^ quartile, Median, Mean, standard deviation, 3^rd^ quartile and maximum of average, standard deviation and Median of chosen delays (SOAs), as well as of confidence, inter-beat interval (in s), localisation distance from the heart and localisation dispersion.

| SOAs | β (Log odds) | *SE* | *z* value | *p*-value | 2.5% | 97.5% | *BF* |
| --- | --- | --- | --- | --- | --- | --- | --- |
|  |  |  |  |  |  |  |  |
| 0 (Intercept) | -0.01 | 0.14 | -0.05 | .961 | -0.20 | 0.20 | 0.01 |
| 100 | 0.33 | 0.10 | 3.32 | .001 | 0.14 | 0.49 | 0.51 |
| 200 | 0.59 | 0.11 | 5.27 | < .001 | 0.40 | 0.76 | 252.44 |
| 300 | 0.49 | 0.13 | 3.82 | < .001 | 0.31 | 0.67 | 76.25 |
| 400 | 0.46 | 0.15 | 3.07 | .002 | 0.28 | 0.64 | 69.53 |
| 500 | 0.29 | 0.18 | 1.63 | .104 | 0.11 | 0.46 | 0.29 |

**Table S4 Mixed-effects regression model to predict the effect of delays (SOAs) on probability to say “yes”**, with random intercepts, including all participants. For each level of the parameter, log odd, standard error (*SE*), *z*-value, *p*-value, 95% confidence interval and Savage-Dickey density ratio Bayes Factor (*BF*) are presented.

(Model_accuracy <- glmer(Jud ~ SOAsF + (1 | pxID), data = dat_long, family = binomial, control = glmerControl (optimizer="bobyqa", optCtrl = list(maxfun = 100000)));

Model_accuracy <-brm(Jud ~ SOAsF +(1|pxID),data =dat_long, family=bernoulli("logit"), prior = set_prior('normal(0,10)'), iter = 2000, chains=4).

N.B.: Log odds values (beta) can be converted back into proportions using the inverse logit formula Exp(beta)/(1+ exp(beta))

| SOAs | β | SE | df | t value | p-value | 2.50% | 97.50% | BF |
| --- | --- | --- | --- | --- | --- | --- | --- | --- |
| (Intercept) | 61.04 | 2.74 | 56.00 | 22.26 | < .001 | 55.62 | 66.46 | >1000 |
| SOAs 100 | -0.40 | 0.91 | 6169.00 | -0.44 | .658 | -2.18 | 1.38 | >1000 |
| SOAs 200 | -2.65 | 0.91 | 6169.00 | -2.91 | .004 | -4.43 | -0.87 | >1000 |
| SOAs 300 | -3.65 | 0.91 | 6169.00 | -4.01 | < .001 | -5.43 | -1.87 | >1000 |
| SOAs 400 | -3.10 | 0.91 | 6169.00 | -3.41 | .001 | -4.88 | -1.32 | >1000 |
| SOAs 500 | -3.75 | 0.91 | 6169.00 | -4.12 | < .001 | -5.53 | -1.97 | >1000 |

**Table S5 Mixed-effects regression model predicting the effect of delays (SOAs) on confidence ratings**, with random intercepts, including all participants. For each level of the parameter, log odd, standard error (*SE*), degree of freedom (*df*), *t*-value, *p*-value, 95% confidence interval and Savage-Dickey density ratio Bayes Factor (*BF*) are presented.

(Model Confidence <- lmer(Conf ~ SOAsF + (1 |pxID), data = dat_long, control = lmerControl(optimizer="bobyqa", optCtrl = list(maxfun = 100000)));

Model Confidence <- brm(Conf ~ SOAsF + (1 |pxID), data = dat_long, family=gaussian , prior = set_prior('normal(0, 10)', class = 'b'), sample_prior = TRUE,iter = 10000, chains=4, save_all_pars = TRUE)).

**Where do people feel their heartbeat and how confident are they?**

| Cluster | β | SE | z.value | *p*.value | 2.50% | 97.50% | BF |
| --- | --- | --- | --- | --- | --- | --- | --- |
| Left part of the chest (Intercept) | 0.26 | 0.10 | 2.59 | 0.010 | 0.06 | 0.46 | 0.26 |
| Left part of the head/ear/neck | -0.12 | 0.12 | -0.98 | 0.328 | -0.35 | 0.12 | 0.02 |
| Right part of the head/ear/neck | 0.37 | 0.11 | 3.29 | 0.001 | 0.15 | 0.59 | 720.06 |
| Right part of the chest | 0.48 | 0.14 | 3.38 | 0.001 | 0.21 | 0.77 | 273.05 |
| Left fingers | 0.25 | 0.18 | 1.40 | 0.163 | -0.10 | 0.61 | 0.43 |
| Miscellaneous | -0.03 | 0.17 | -0.16 | 0.874 | -0.36 | 0.31 | 0.03 |
| Right fingers | -0.47 | 0.27 | -1.65 | 0.098 | -1.04 | 0.08 | 0.02 |
| Left arm | -0.31 | 0.39 | -0.79 | 0.431 | -1.09 | 0.46 | 0.03 |

**Table S6 Mixed-effects regression model predicting the effect of spatial clusters on simultaneity judgements,** with random intercepts, including participants with localisation data (N=50). For each level of the parameter, log odd, standard error (*SE*), *z*-value, *p*-value, 95% confidence interval and Savage-Dickey density ratio Bayes Factor (*BF*) are presented.

(Model Accuracy Cluster <- glmer(Jud~ cluster_newF + (1 |pxID), dat_long2, family= binomial, control = glmerControl(optimizer="bobyqa", optCtrl = list(maxfun = 100000)));

Model Accuracy Cluster <- brm(Jud~ cluster_newF + (1 |pxID), data = dat_long2, family=bernoulli("logit"), prior = set_prior('normal(0, 10)', class = 'b'), sample_prior = TRUE,iter = 2000, chains=4, save_all_pars = TRUE).

N.B.: Log odds values (beta) can be converted back into proportions using the inverse logit formula Exp(beta)/(1+ exp(beta))

| Cluster | β | SE | df | t.value | *p*.value | 2.50% | 97.50% | BF |
| --- | --- | --- | --- | --- | --- | --- | --- | --- |
| Left part of the chest (Intercept) | 55.75 | 2.52 | 54.32 | 22.10 | <.001 | 50.78 | 60.73 | >1000 |
| Left part of the head/ear/neck | 8.155 | 1.30 | 5709.29 | 6.25 | <.001 | 5.6 | 10.70 | >1000 |
| Right part of the head/ear/neck | 5.70 | 1.17 | 5825.64 | 4.89 | <.001 | 3.41 | 7.98 | >1000 |
| Right part of the chest | 6.33 | 1.51 | 5715.00 | 4.21 | <.001 | 3.39 | 9.28 | >1000 |
| Left fingers | 3.42 | 1.88 | 5883.16 | 1.82 | .068 | -0.27 | 7.09 | >1000 |
| Miscellaneous | 1.14 | 1.77 | 5888.50 | 0.65 | .518 | -2.33 | 4.60 | >1000 |
| Right fingers | 5.32 | 3.18 | 5572.23 | 1.68 | .094 | -0.90 | 11.53 | >1000 |
| Left arm | 7.16 | 4.46 | 5363.83 | 1.60 | .109 | -1.57 | 15.92 | >1000 |

**Table S7 Mixed-effects regression model predicting the effect of spatial clusters on confidence ratings,** with random intercepts, including participants with localisation data (N=50). For each level of the parameter, log odd, standard error (SE), degree of freedom (df), t-value, p-value, 95% confidence interval and Savage-Dickey density ratio Bayes Factor (BF) are presented.

(Model Confidence Cluster <-lmer(Conf~ cluster_newF + (1 |pxID), dat_long2, family= binomial, control =lmerControl(optimizer="bobyqa", optCtrl = list(maxfun = 100000)));

Model Confidence Cluster <- brm(Jud~ cluster_newF + (1 |pxID), data = dat_long2, family=bernoulli("logit"), prior = set_prior('normal(0, 10)', class = 'b'), sample_prior = TRUE,iter = 2000, chains=4, save_all_pars = TRUE)

***Individual performances***


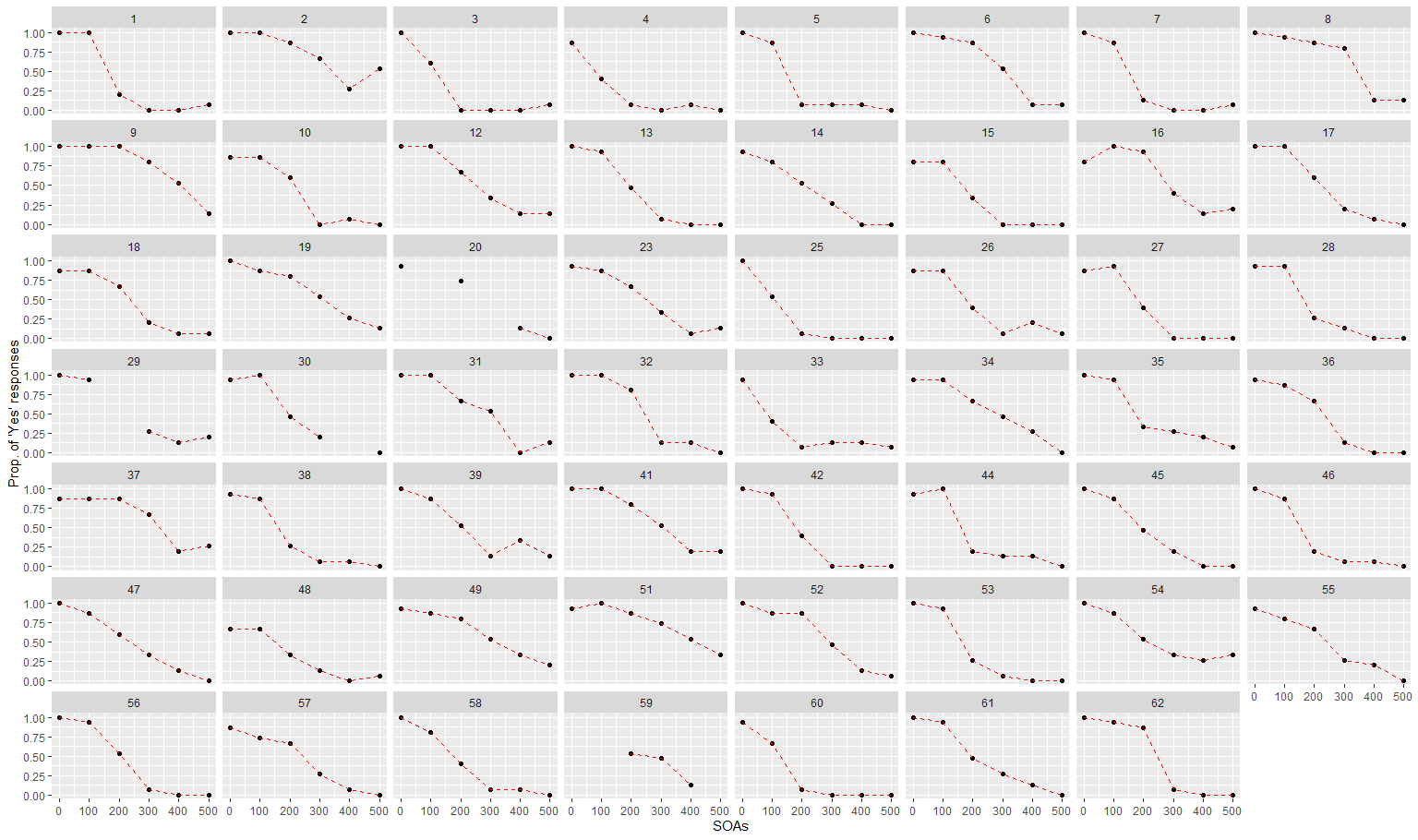


Figure S5: Individual performances of participant on the audio-visual simultaneity task (N=55, after removing 7 outliers)


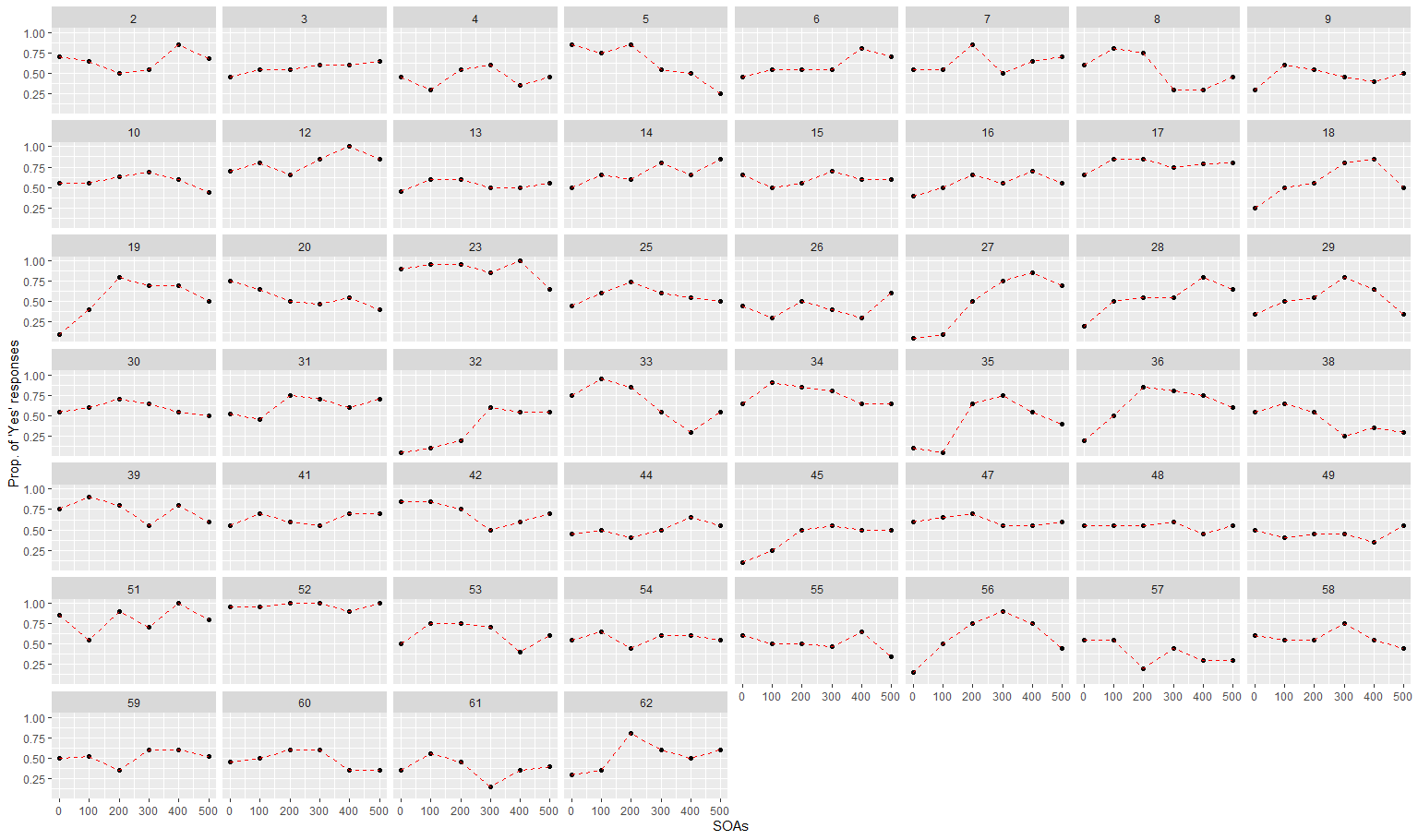


Figure S6 Temporal perception of heartbeat sensation for each participant. (N=52, after removing 10 outliers)


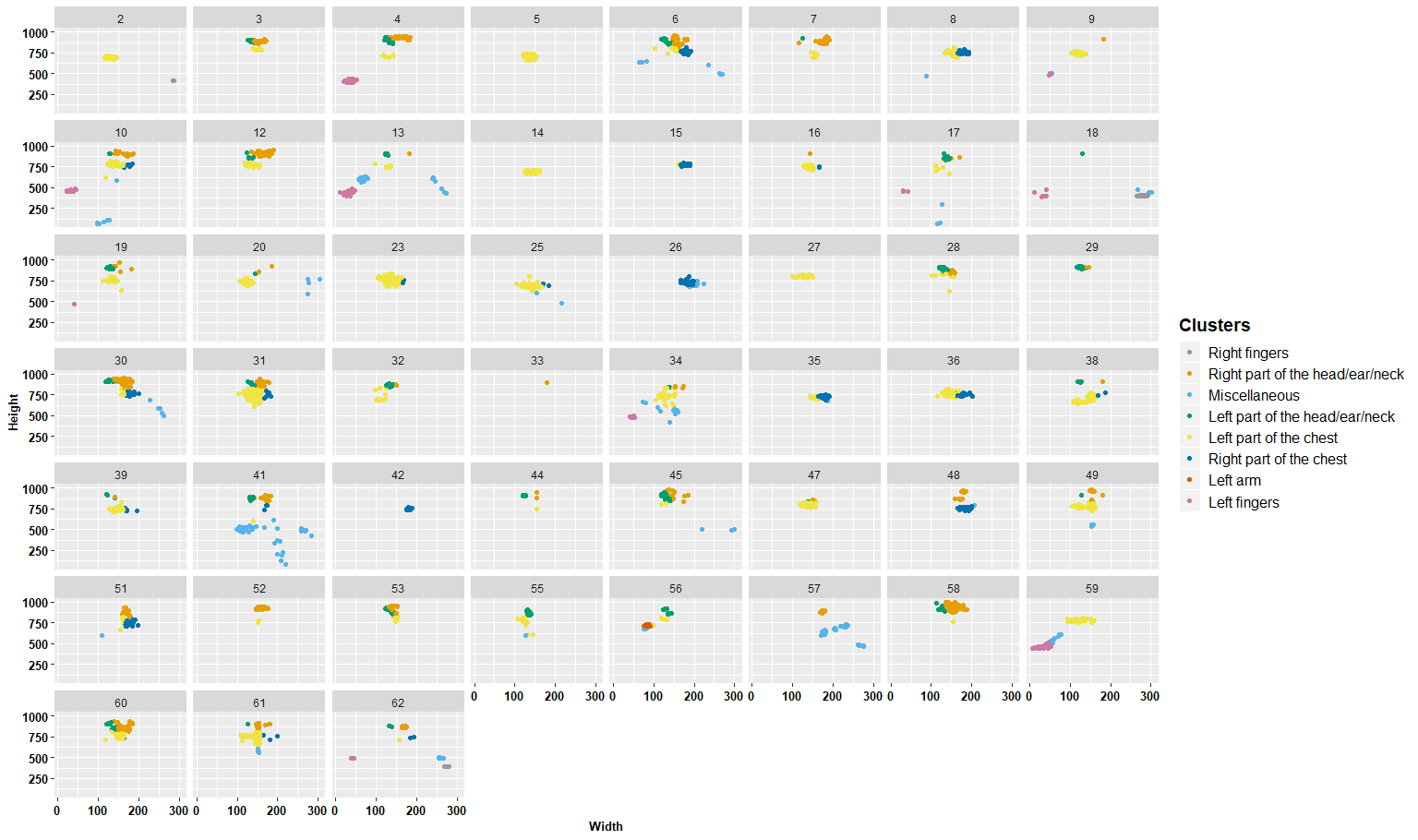


Figure S7 Clusters and sampling dispersion for each participant (N=51, after removing 10 outliers, and px54 who did not have heartbeat localisation data due to a technical issue Px33 who is represented on the graph only had one data point of heartbeat localisation data due to a technical issue and was discarded from the heartbeat localisation analyses.)
